# Being Passionate to Perform: The Joint Effect of Leader Humility and Follower Humility

**DOI:** 10.3389/fpsyg.2019.01059

**Published:** 2019-05-14

**Authors:** Huiyue Diao, Lynda Jiwen Song, Yue Wang, Jun Zhong

**Affiliations:** ^1^Department of Organization and Human Resources, School of Business, Renmin University of China, Beijing, China; ^2^Department of Management and Marketing, The Hong Kong Polytechnic University, Kowloon, Hong Kong

**Keywords:** leader humility, follower humility, job performance, harmonious passion, self-determination theory, China

## Abstract

Although humility is an outstanding characteristic of many beloved and respected leaders, little is understood regarding the effect of leader humility on follower job performance. The current study examines how leader humility affects follower performance. Drawing on the self-determination theory, we suggest that leader humility, via follower harmonious passion, contributes to follower performance. With multiphase leader-follower paired data, we find that leader humility is positively related to follower performance, this positive relationship is partially mediated by follower harmonious passion, and the indirect effect of leader humility on follower performance via follower harmonious passion is stronger with a high level of follower humility.

## Introduction

Treated as an essential characteristic of leaders by scholars (Collins, 2001; Vera and Rodriguez-Lopez, 2004; Morris et al., 2005; Owens et al., 2013; Oc et al., 2015), humility has been defined as an interpersonal characteristic, and expressed humility implies “(a) a manifested willingness to view oneself accurately, (b) a displayed appreciation of others’ strengths and contributions, (c) teachability” (Owens et al., 2013, p. 1518), which is also known as openness to feedback, advice, and new ideas (Rego et al., 2017). Rather than being a sign of self-abasement, low self-esteem, lack of confidence, or lack of ambition, humility is a virtue (Tangney, 2000; Exline and Geyer, 2004; Vera and Rodriguez-Lopez, 2004). With self-awareness, transcendence, and openness being humility’s three dimensions (Morris et al., 2005), humility offers a leader competitive advantage by furnishing him/her with a realistic perspective of himself/herself, a proper assessment of success and failure, and a down to earth evaluation of the events and relationships in his/her work and life (Vera and Rodriguez-Lopez, 2004).

Leaders have been regarded as an important contextual factor on followers’ work attitudes and behaviors (Morris et al., 2005; Perry et al., 2010). In this study, following organizational behavior literatures (such as Exline and Geyer, 2004; Nielsen et al., 2010; Owens et al., 2013; Ou et al., 2017; Rego et al., 2017), a “leader” means a director who is in charge of a particular group, team, department or organization, or a supervisor of a certain group of people. A “follower” here means a subordinate who has a lower position in an organization than his/her leader. Researchers in other fields may have different understandings of “leader” and “follower,” such as Nakayama et al. (2017), focusing on the behavioral phenotype of individuals.

The influences of leader humility on their subordinates’ attitudes and behaviors are attracting more attention. Previous studies on leader humility find that it has positive relations with follower engagement (Owens et al., 2013; Sousa and Dierendonck, 2017), follower identification with leader, follower trust in leader, follower self-efficacy, follower motivation (Nielsen et al., 2010), and follower job satisfaction (Owens et al., 2013; Ou et al., 2017). Scholars find that leader humility contributes to climate changes which benefit collective performance, such as companies’ long-term performance, influenced by company leaders’ humility via building a more collaborative environment (Mayo, 2017), and team performance, raised by team leaders’ humility via increasing team collective humility and collective promotion focus (Owens and Hekman, 2016). However, the underlying psychological mechanism about how followers process the influence of leaders’ humility on an individual level, leading to a rise in performance remains unclear. Considering that the relationship between leader humility and team member performance is still lacking direct evidence, and the effective process remains unclear, this study tries to uncover the mechanism between leader humility and follower performance from a follower perspective.

According to self-determination theory, employees have “three innate psychological needs—competence, autonomy, and relatedness” (Ryan and Deci, 2000, p. 68), which facilitate intrinsic work motivation (Gagné and Deci, 2005), with which one’s performance is more likely benefited by initiatives (Grant et al., 2011). Therefore, self-determination theory offers a clue of why followers, perceiving leader humility, may improve their performance.

We would like to suggest a mediating mechanism of leader humility on followers’ performance via their harmonious passion. Passion refers to “a strong inclination toward an activity that people like, that they find important, and in which they invest time and energy” (Vallerand et al., 2003, p. 756). Harmonious passion, as a motivational mechanism offering a better motivational quality than extrinsic motivation or intrinsic motivation (Liu et al., 2011), leads a person to willingly engage in an activity (Bélanger et al., 2013), which is autonomously internalized within one’s identity (Vallerand et al., 2003; Forest et al., 2012), with pleasure and enjoyment. It has been proposed to be positively related to employee positive emotions during activity engagement, quality of interpersonal relationships (Philippe et al., 2010), well-being, work satisfaction (Carbonneau et al., 2008; Vallerand et al., 2010), and performance (Bonneville-Roussy et al., 2011; Ho et al., 2011; Dubreuil et al., 2014; Astakhova and Porter, 2015). In this study, by self-determination theory, we would like to propose a motivational mechanism that a humble leader, good at facilitating the fulfillments of followers’ competence, autonomy, and relatedness needs, contributes to followers’ job performance by increasing their harmonious passion toward jobs.

In summary, by the self-determination theory, the main purposes of our study are to examine the link between leader humility and follower performance, and to discover the role of follower harmonious passion and follower humility within this. This study aims to contribute to leadership and passion literature in three ways. (1) Although several studies have examined leader humility’s influence on performance by focusing mainly on collective mechanism (Ou et al., 2014; Owens and Hekman, 2016), they somehow leave a gap of inquiry as to how the influence of leader humility is processed by an individual’s psychological mechanism. We contribute to leader humility literature by showing that leader humility benefits follower performance via the psychological mediating mechanism of harmonious passion. (2) We extend the leadership effectiveness literature by taking into account a follower characteristic, which indicates not only how comfortable followers are with leader behaviors but also how followers judge those behaviors. Considering that different persons have different opinions on humility as a characteristic of leaders (Exline and Geyer, 2004), we examine the moderating role of follower humility. (3) While most previous leader humility scholars focus on role modeling effects (e.g., Nielsen et al., 2010; Oc et al., 2015; Mayo, 2017), our work is among the first to link leader humility theories and self-determination theory together. We explore an alternative explanation of how leader humility benefits follower outcomes to highlight leader humility as a follower work passion trigger.

## Leader Humility and Follower Performance

Our study follows Owens et al. (2013) definition of expressed humility. A humble leader expresses humility through three kinds of humble behaviors: admitting mistakes and limitations, spotlighting follower strengths and contributions (Owens and Hekman, 2012), and being open to learning, feedback, and new ideas (Owens et al., 2013).

All three dimensions may work in a self-determination process to benefit follower performance by supporting followers’ work competence and autonomy, given that support for competence and autonomy facilitates their motivation and human growth (Ryan and Deci, 2000). First, a humble leader acknowledges him/herself as having limitations and wants to have an accurate view on him/herself (Mayo, 2017). In this case, followers are allowed to make judgments on the leader and the leader’s suggestions or proposals, and they do not feel their critical opinions forbidden. Therefore, in the first dimension of leader humility, follower autonomy is protected. Second, a follower feels that his/her competence has been acknowledged having had a leader who points out his/her strengths and highlights their contributions. This follower then has more confidence in his/her job, and more motivation stimulated (Gerhart and Fang, 2015). Thus, this follower has more initiative to be productive (Grant et al., 2011), and is more likely to have a rise in performance. Third, according to self-determination theory, the third dimension contributes to followers’ experience of work autonomy due to the fact that a humble leader is open to new ideas and ways (Owens et al., 2013), and followers are encouraged to make suggestions, generate new ideas, and be creative in their work processes. Thus, in an autonomy-supportive condition, followers’ effective performance can be promoted (Gagné and Deci, 2005; Grant, 2008; Grant et al., 2011).

### Mediating Role of Follower Harmonious Passion

To go further with the underlying mechanism, our study theorizes that harmonious passion plays a mediating role in the link between leader humility and follower performance. Harmonious passion, resulting from an autonomous internalization of an activity into a person’s identity (Vallerand et al., 2007), is a strong but controllable desire to engage in an activity (Bélanger et al., 2013). With this kind of passion, a person freely decides whether to engage in an activity or not (Bonneville-Roussy et al., 2011), meaning this passionate pursuit of an activity comes together in a harmonious way with other aspects of the person’s life (Vallerand et al., 2008). The development of harmonious passion is positively influenced by autonomy support from a parent or a significant adult (Mageau et al., 2009; Forest et al., 2012), or contextual autonomy support (Liu et al., 2011). Autonomy is related to acting with the experience of choice, a sense of volition, and a high level of reflection (Dworkin, 1988; Gagné and Deci, 2005). A leader with humility, as a significant person to his/her followers and creating a significant environmental condition in the workplace, may express his/her autonomy support to his/her followers in three ways.

First, willingness to judge him/herself fairly (Tangney, 2000) and to have a balanced view (Mayo, 2017), accepting the fact that everyone has weaknesses and limitations (Clark, 1992). A leader sends out signals signaling that reasonable criticism is acceptable and welcome in this team (Morris et al., 2005), and that followers are allowed to have their own judgments of how things should be done and what can make a better leader. That means, instead of being forced to agree with everything, team members, having a humble leader, tend to have choices when engaging with and judging things in their work. In this way, perceiving a leader’s humility, team members may feel that they have a leader offering them freedom of judgment and supporting their autonomy.

Second, appreciating and acknowledging followers’ strengths and contributions (Oc et al., 2015), a leader with humility shows his/her confidence in followers’ capabilities (Nielsen et al., 2010) and, with this confidence, the leader tends to enact more empowering behaviors (Ou et al., 2014). Being trusted with competencies and empowered by the leader, followers may experience more autonomy when they make decisions on what to do, and how to do, at work. Thus, perceiving a leader’s humility, team members may get more autonomy support from the leader with more empowering behaviors.

Third, open to advice, new ideas, information, and feedback (Vera and Rodriguez-Lopez, 2004; Owens et al., 2013), a humble leader has a habit of listening before speaking (Owens and Hekman, 2012). Perceiving a leader as such a good “learner” (Vera and Rodriguez-Lopez, 2004), followers may believe their suggestions are treated conscientiously and fairly. They are then encouraged to think, voice, and be creative for implementation (Liu et al., 2017), during which they may experience successive feelings of making decisions and choices, enhancing their feeling of autonomy. That is, perceiving a leader’s humility, followers may find autonomy support from the leader and stimulate their own harmonious passion, which may previously be undiscovered or hidden.

Motivation is about many aspects of activation and intention, such as energy, direction, and persistence (Ryan and Deci, 2000). With a stronger motivation regarding his/her job, a person more devoutly engages in his/her work (Zhang et al., 2018) and is likely to perform better. Harmonious passion is a motivation allowing a person to freely and autonomously engage in an activity with joy, pleasure, and low level pressure (Vallerand et al., 2003). A person’s harmonious passion on his/her job therefore will be positively related to goal pursuing and job performance (Vallerand et al., 2008; Bonneville-Roussy et al., 2011).

Perceiving a leader’s humility, a team member may feel more autonomy support from the leader, and more freely experience choices and decisions with less pressure. Accomplishing tasks in a way that fits his/her identity, more harmonious passion for his/her job may be stimulated. With a higher level of harmonious passion at work, and being better motivated (Forest et al., 2012), he/she is more likely to put forth additional effort that results in better performance. Thus, we hypothesize:

*Hypothesis 1: Follower harmonious passion mediates the positive relation between leader humility and follower performance*.

### Moderating Role of Follower Humility

Although humility is a virtue (Tangney, 2000; Exline and Geyer, 2004) bringing a leader competitive advantage (Vera and Rodriguez-Lopez, 2004), it is viewed in a different way when some dictionary definitions of humility are associated with self-abasement (Exline and Geyer, 2004). Even those who believe humility is a positive characteristic may value leader humility differently. Exline and Geyer (2004) find many of their research participants do not think humility is a characteristic that a leader should have.

For the relationship between leader humility and follower harmonious passion, a key condition is how the followers judge leader humility as a leader’s interpersonal behavior characteristic: Will a follower accept leader humility as a beneficial characteristic which offers follower autonomy support, or a sign of someone weak or lacking confidence which sends out the message that the leader is not qualified or powerful enough?

Compared with “superhero” kinds of leaders, such as charismatic or narcissistic leaders, leaders with humility are like unsung heroes (Mayo, 2017). To make their leadership effective, unsung heroes need their “soul mates” to detect their beauties and strengths, and to utilize and carry forward those strengths properly.

When a follower behaves humbly, it is more likely for him/her to appreciate others’ humility as a virtue, considering that the follower approves the advantages of behaving humbly or the merits of being humble. For example, such a follower may have a better understanding of the opportunities offered by a leader with humility and feel more comfortable in the environment created by this leader than other team members who prefer clear orders, instead of being asked to behave independently and creatively. A follower with humility, being the same kind of person as the leader in this sense, is more likely to sense a humble leader’s autonomy support and be more comfortable in an autonomy supportive context, meaning more harmonious passion at work is stimulated. Thus, we hypothesize:

Hypothesis 2: Follower humility positively moderates the relationship between leader humility and follower harmonious passion, such that the positive relation between leader humility and follower harmonious passion is stronger when follower humility is high versus low.

We have hypothesized the positive relationship between leader humility and follower performance, the positive relationship between leader humility and follower harmonious passion at work, a mediating process linking leader humility and follower performance with follower harmonious passion, and a moderating role of follower humility in the relationship between leader humility and follower harmonious passion. To combine these hypotheses, we raise an integrative moderated mediation model, suggesting that when a follower is of high level humility, perceiving leader humility will lead him/her to sense more autonomy support and feel more comfortable than those of low level humility, causing a greater amount of harmonious passion of him/her at work to be stimulated and contributing to stronger performance improvement. Thus, we hypothesize:

Hypothesis 3: Follower humility positively moderates the indirect positive relation between leader humility and follower performance via follower harmonious passion, such that the indirect effect of leader humility on follower performance is stronger when follower humility is high versus low.

**FIGURE 1:**
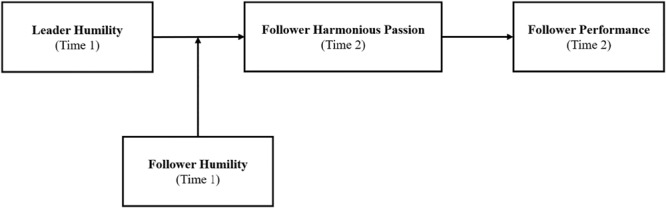
Theoretical model.

Figure 1 demonstrates the proposed theoretical model in this study.

## Methods

### Participants and Procedures

We surveyed 214 employees within 52 work teams from a Chinese high-tech company, during June and July 2017. To test our hypotheses, we designed two versions of questionnaires: a follower version for team members and a leader version for their immediate supervisors, the team leaders. We collected data in two steps: (1) at Time 1, we sent out 214 follower and 52 leader questionnaires and received completed usable responses from 206 followers and 50 leaders (response rates 96.3% and 96.2%, respectively); and (2) 1 month later, we sent out Time 2 questionnaires to those who completed the Time 1 survey, and received completed usable responses from 200 followers and 50 leaders (response rates 97.1% and 100%, respectively). With the two-step data collection, we obtained the data of 200 leader-follower pairs. The final sample was mostly of males (70%), with an average age of 28.8 years (*SD* = 4.21). The followers’ average team tenure was 26 months (*SD* = 31.25).

In the Time 1 survey, we collected follower age, follower gender, follower team tenure leader humility, follower harmonious passion, and follower humility in the follower version questionnaires, and leaders were asked to fill in their age, gender, and team tenure. One month later, in the Time 2 survey, we used follower version questionnaires to assess follower harmonious passion again, and used leader version questionnaires to let leaders assess their followers’ performance correspondingly.

We conducted the study with this Chinese high-tech company for the following four reasons. First, a China sample could be a very appropriate sample, because it is likely for the participants to experience leaders’ humble behaviors, given that humility is in line with Chinese social value system meaning leaders may behave humbly. As President Xi (2014) stated, one should always be humble and prudent. Second, due to China’s frequent international interactions through which and Eastern and Western cultures are fused together in the society (Ou et al., 2014), a China sample could also be representative of the way in which humility is broadly valued in both East and West societies (Peterson and Seligman, 2004). Third, with China’s industry development, many competitive companies are technology-driven, and healthily growing high-tech companies are representative of this. Fourth, employees in this company came from all over China, which could facilitate the overall sample results, as there is little bias of specific local cultures.

### Measures

In this study, all survey measures, excepting the demographic variables, were scaled to a seven-point scale, ranging from 1 to 7 (1 = strongly disagree; 7 = strongly agree). For all used scales, one member of our research team first translated the English scales into Chinese, another member translated these versions back into English, and a third member compared two versions of English scales and readjusted the translation where the meanings were inconsistent.

#### Leader Humility

Leader humility was measured with a 9-item other-report scale (Owens et al., 2013). We used this scale as Owens et al. (2013) paper was among the first to propose the concept of expressed humility and gave a scale for measuring one’s humility by behavior; this scale was used with high reliabilities in several previous studies, such as Basford et al. (2014) research, Jeung and Yoon (2016) study, Rego et al. (2017) work, Qian et al. (2018) study. In this study, leader humility was reported by followers due to two considerations: first, before sending out questionnaires, members of our research team interviewed several team leaders and followers in this company and during these interviews we noticed leaders tend to report themselves as humble persons, even when their followers sometimes held different ideas supported by proved cases; second, with different followers, a leader may express humility in different degrees or different ways (Rego et al., 2017). Example items in the scale are “My leader actively seeks feedback even if it is critical,” “My leader takes notice of others’ strengths,” and “My leader is willing to learn from others.” This variable was assessed by followers at Time 1 (α = 0.93).

#### Follower Humility

Follower humility was also assessed by Owens et al. (2013) 9-item scale. This scale was used to be consistent with leader humility in this study, however, considering the focus of this study we adapted this peer-report scale to a self-report one. By self-reporting, one’s answer not only reflects the level of his/her humble behaviors but also reveals the degree of a follower’s agreeing to such behaviors. Example items in the scale are “I acknowledge when others have more knowledge and skills than me,” “I show appreciation for the unique contributions of others,” and, “I am open to the advice of others.” This variable was assessed by followers at Time 1 (α = 0.84).

#### Follower Harmonious Passion

We used Vallerand et al. (2003) 7-item self-report scale to measure follower harmonious passion. This scale was used in this study because Vallerand et al. (2003) study was among the most influencing discussing passion at work and proposed that harmonious passion and obsessive passion were of differences; it was broadly used by passion researchers, such as Vallerand et al. (2007); Ho et al. (2011), Liu et al. (2011), and Astakhova and Porter (2015). Example items in the scale are, “This activity allows me to live a variety of experiences,” “The new things that I discover with this activity allow me to appreciate it even more,” and, “This activity is in harmony with the other activities in my life.” This variable was assessed by followers at both Time 1 (as a control variable) and Time 2 (α = 0.85).

#### Follower Performance

Follower performance was measured with Van Dyne and LePine’s (1998) 4-item other-reported scale for in-role performance. We used this scale as it is a classic scale for work performance and clearly separated the extra-role performance and in-role performance (Griffin et al., 2007); furthermore, it had a high reliability with supervisor-rated (see Van Dyne and LePine, 1998). Example items in the scale are, “This particular employee fulfills the responsibilities specified in his/her job description,” and, “This particular employee performs the tasks that are expected as part of the job.” This variable was assessed by team leaders at Time 2 (α = 0.93).

#### Control Variables

In this study, we controlled follower age, follower gender, follower team tenure, leader age, leader gender, leader team tenure, and Time 1 follower harmonious passion. We controlled follower age and leader age because people of different generations may value humility differently, especially with the consideration that Chinese society has experienced several significant changes during the previous four decades. Follower gender and leader gender were controlled due to previous studies suggesting female members are more responsive to humble behaviors (Owens and Hekman, 2016). Follower and leader team tenure were controlled, because the time that they have spent together may relate to how much influence a member gets from a leader. While Forest et al. (2012) suggest that harmonious passion is not a state of mind but a self-defining characteristic, Liu et al. (2011) have demonstrated it being influenced by environmental conditions. Hence, to test whether a part of followers’ harmonious passion is stimulated by others’ behaviors, leaders’ humble behaviors, and how that changed part bridges the relationship between leader humility and one’s performance, we controlled Time 1 follower harmonious passion.

### Analytical Methods

Before the hypotheses testing, a set of confirmatory factor analyses (CFAs) were conducted to test the discriminant validity of factors in the proposed model – whether the proposed four-factor model is better than more parsimonious models: a three-factor model, a two-factor model and a one-factor model. With a hypothesized model, a CFA is to compare the difference between an estimated covariance matrix and the observed covariance matrix (Schreiber et al., 2006). The smaller the difference is, the better. Various goodness-of-fit indicators are used by scholars and some popular indicators are NFI, IFI, TLI, CFI, RMSEA (Hu and Bentler, 1999). A good fit normally requires NFI, IFI, TLI, and CFI equal to or higher than 0.95 and RMSEA lower than 0.08 (Schreiber et al., 2006).

The first step of hypotheses testing in this study was to test the mediating effect (Hypothesis 1). One of the most popular logics of mediation testing consists of three steps suggested by Baron and Kenny (1986): (1) the independent variable (X) affects the dependent variable (Y); (2) the X affects the mediator (M); and (3) controlling M, the effect of X on Y no longer exists or becomes less than that in the first step. Correspondingly, three regression equations, Eqs. (1–3), can be built.

(1) $$\begin{array}{c} Y=\beta_{01}+\beta_{1}X+\varepsilon_{1}\;\;\;\;\;\;\;\;\left(\varepsilon \;\mathrm{is}\;\mathrm{random}\;\mathrm{error}\right) \end{array}$$

(2) $$\begin{array}{c} M=\beta_{02}+\beta_{2}X+\varepsilon_{2} \end{array}$$

(3) $$\begin{array}{c} Y=\beta_{03}+\beta_{3}X+\beta_{4}M+\varepsilon_{3} \end{array}$$

With a regression module in SPSS, results indicating a significant mediation are: in the first and second model, none of the coefficients for X (*β_1_, β_2_*) are equal to 0, and *p*-values for the coefficients (*β_1_, β_2_*) are both below 0.05 (significant) or below 0.10 (marginal significant); in the third model, the coefficient for X (*β_3_*) is equal to 0 or less than that in the first model (*β_1_*), meanwhile the coefficient for M (*β_4_*) is not equal to 0 and its *p*-value meets the significance requirement.

To test the moderation (Hypothesis 2), considering that both the independent variable (X) and the moderator (W) are continuous variables, this study followed Luo and Jiang’s (2012) suggestion building a regression equation as Eq. (4).

(4) $$\begin{array}{c} Y=\beta_{04}+\beta_{5}X+\beta_{6}W+\beta_{7}Z\left(X\right)\times Z\left(M\right)+\varepsilon_{4} \end{array}$$

Z(X) and Z(Y) are standardized X and standardized Y using Z score, in order to reduce the multicollinearity (Luo and Jiang, 2012). With a regression module in SPSS, results indicating a significant moderation are: *β_7_* ≠0 and *p-*value for *β_7_* is below 0.05 (significant) or 0.10 (marginal significant).

In a mono-level first stage moderated mediation model, such as the model proposed in this study, the moderated mediating effect can be tested with regression equation as Eq. (7), which is a combination of Eq. (5) and Eq. (6) (Liu et al., 2012).

(5) $$\begin{array}{c} M=\beta_{05}+\beta_{8}X+\beta_{9}W+\beta_{10}XW+\varepsilon_{5} \end{array}$$

(6) $$\begin{array}{c} Y=\beta_{06}+\beta_{11}X+\beta_{12}M+\beta_{13}W+\beta_{14}XW+\varepsilon_{6} \end{array}$$

(7) $$\begin{array}{c} \;\;\;\;\;\;\;\;\;Y=\beta_{06}+\beta_{12}\beta_{05}+[\beta_{11}+\beta_{12}\left(\beta_{8}+\beta_{10}W\right)]X+\left(\beta_{13}+\beta_{12}\beta_{9}\right)W+\beta_{14}XW+\varepsilon_{7} \end{array}$$

The key to test the moderated mediation is the differences between indirect effects with W having different values. Therefore, a moderated mediating effect exists when, with a desired confidence interval, [β_12_ (β_8_+β_10_W_High_|β_12_(β_8_+β_10_W_low_)] does not contain 0. As suggested by Edwards and Lambert (2007), a moderated mediation can be tested with a bootstrapping analysis, which allows bootstrap samples being used to locate the upper and lower bounds of the desired confidence interval. PROCESS is a module facilitating bootstrapping analysis in SPSS and SAS and can be used for testing mediation, moderation, mediated moderation and moderated mediation (Hayes, 2013). With the PROCESS module in SPSS, normally, a result indicating a significant effect is that 95% confidence interval [CI] does not contain 0.

Overall, analytical strategy for this study is as follows. After conducting CFAs with AMOS 22, we applied hierarchical regression analyses in SPSS 23 to test the direct effect of leader humility on follower performance, the mediating role of follower harmonious passion (Hypothesis 1), and the moderating role of follower humility (Hypothesis 2). And we run supplementary tests for the mediating effect and the moderating effect with a more rigorous analysis method, bootstrapping analysis (using 20,000 bootstrap samples) with the PROCESS module in SPSS provided by Hayes (2013). Then, we use bootstrapping analysis to test the moderated mediation (Hypothesis 3).

## Results

The variables’ means, standard deviations, reliabilities, correlations, and collection schedule are shown in Table 1. In this study, all variables’ reliabilities are above 0.80, and correlations of tested variables are as expected.

**Table 1:** Means, standard deviations, reliabilities, correlations, and collection schedule.

Variable	*Mean*	*SD*	1	2	3	4	5	6	7	8	9	10	11
1. Follower age (T1)	28.80	4.21	–										
2. Follower gender (T1)	0.30	0.46	–0.08	–									
3. Follower team tenure (T1)	26.00	31.25	0.13	0.03	–								
4. Leader age (T1)	35.30	5.52	0.04	–0.03	–0.03	–							
5. Leader gender (T1)	0.11	0.31	0.04	0.17*	–0.06	–0.04	–						
6. Leader team tenure (T1)	33.26	25.24	–0.01	0.05	0.03	0.30**	0.17*	–					
7. Follower harmonious passion (T1)	5.05	0.95	0.16*	–0.10	–0.07	0.09	0.02	0.01	(0.86)				
8. Leader humility (T1)	5.36	1.04	–0.07	–0.16*	–0.14*	0.01	0.06	–0.11	0.32**	(0.93)			
9. Follower humility (T1)	6.09	0.56	–0.10	–0.01	–0.04	–0.01	0.03	0.01	0.38**	0.40**	(0.84)		
10. Follower harmonious passion (T2)	5.08	0.84	0.23**	–0.07	–0.03	0.07	–0.02	–0.07	0.71**	0.31**	0.31**	(0.85)	
11. Follower performance (T2)	5.78	0.85	0.10	–0.04	0.14	–0.01	–0.01	0.01	0.06	0.18*	0.11	0.17*	(0.93)

*N = 200. Cronbach’s alphas are reported in the parentheses on the diagonal. ^∗∗^*p* < 0.01; ^∗^*p* < 0.05 (two-tailed).*

### Preliminary Analysis

The CFA results for our four-factor model and the alternative models are summarized in Table 2. Results reveal satisfactory fit for the four-factor model (leader humility, follower humility, follower harmonious passion, and follower performance): χ^2^/*df* = 1.86, NFI = 0.95, IFI = 0.98, TLI = 0.97, CFI = 0.98, and RMSEA = 0.07. The four-factor model has significantly better fit than: a three-factor model, where leader humility and follower harmonious passion items loaded on one factor and all other variables on separate factors (χ^2^/*df* = 7.80, NFI = 0.78, IFI = 0.80, TLI = 0.74, CFI = 0.80, and RMSEA = 0.19); a two-factor model, where leader humility and follower harmonious passion items loaded on one factor and all other items on a second factor (χ^2^/*df* = 17.38, NFI = 0.47, IFI = 0.47, TLI = 0.38, CFI = 0.48, and RMSEA = 0.29); and a one-factor model (χ^2^/*df* = 22.33, NFI = 0.30, IFI = 0.31, TLI = 0.19, CFI = 0.30, and RMSEA = 0.33). The CFA results demonstrate acceptable discriminant validity of the four variables.

**Table 2:** Confirmatory factor analysis.

Model Factor	χ^2^	*/df*	NFI	IFI	TLI	CFI	RMSEA
Four-factor model	1.86	0.95	0.98	0.97	0.98	0.07
Three-factor model: leader humility and follower harmonious passion combined	7.80	0.78	0.80	0.74	0.80	0.19
Two-factor model: leader humility and follower harmonious passion combined; follower performance and follower humility combined	17.38	0.47	0.47	0.38	0.48	0.29
One-factor model	22.33	0.30	0.31	0.19	0.30	0.33
**Decision value of each index**	<5	>0.9	>0.9	>0.9	>0.9	<0.08

*N = 200.*

### Hypotheses Testing

Hypothesis 1 proposes that follower harmonious passion mediates the positive relationship between leader humility and follower performance. We first followed Baron and Kenny’s (1986) procedures to test this hypothesis with hierarchical regression analysis. As shown by the results of model 2 in Table 3, leader humility (T1) is positively related to follower performance (T2) (*β* = 0.18, *p* < 0.01). As demonstrated by the results of model 5 in Table 3, leader humility (T1) is positively related to follower harmonious passion (T2) (*β* = 0.09, *p* < 0.05). As indicated by the results of model 3 in Table 3, when follower harmonious passion (T2) is added as a predictor of follower performance (T2), follower harmonious passion (T2) is positively related to follower performance (T2) (*β* = 0.20, *p* < 0.10) and the significance level of leader humility (T1) is lowered (*β* = 0.16, *p* < 0.05). We then retested the mediation with bootstrapping analysis. As shown by the results in Table 4, the indirect effect of leader humility on follower performance via follower harmonious passion is significant (95% confidence interval [CI] = 0.0013, 0.0551 [not containing 0]). Therefore, Hypothesis 1 is supported.

**Table 3:** Regression results for follower harmonious passion and performance.

Variable	Follower performance (T2)			Follower harmonious passion (T2)			
	Model 1	Model 2	Model 3	Model 4	Model 5	Model 6	Model 7
Intercept	5.09**	4.25**	4.09**	1.25**	0.82+	0.50	0.28
Follower age (T1)	0.02	0.02	0.02	0.02*	0.03**	0.03**	0.03**
Follower gender (T1)	–0.06	–0.01	–0.11	0.02	0.05	0.05	0.05
Follower team tenure (T1)	0.01+	0.01*	0.01*	0.01	0.01	0.01	0.01
Leader age (T1)	–0.01	–0.01	–0.01	0.01	0.01	0.01	0.01
Leader gender (T1)	–0.01	–0.07	–0.05	–0.06	–0.09	–0.09	–0.11
Leader team tenure (T1)	0.01	0.01	0.01	–0.01	–0.01	–0.01	–0.01
Follower harmonious passion (T1)	0.05	–0.01	–0.13	0.61**	0.58**	0.57**	0.55**
Leader humility (T1)		0.18**	0.16*		0.09*	0.08+	0.08+
Follower harmonious passion (T2)			0.20+				
Follower humility (T1)						0.07	0.10
Leader humility (T1)^∗^follower humility (T1)							0.07+
*R^2^*	0.03	0.07+	0.09*	0.52**	0.53**	0.53**	0.54**
*F*	0.88	1.79+	2.05*	29.99**	27.21**	24.20**	22.27**
*ΔR^2^*	0.03	0.04**	0.02*	0.52**	0.01*	0.01	0.01+
*ΔF*	0.88	7.93**	3.91*	29.99**	4.23*	0.58	2.81+

*N = 200. ^+^*p* < 0.10; ^∗^*p* < 0.05; ^∗∗^*p* < 0.01 (two-tailed).*

**Table 4:** Bootstrapped effects on follower performance.

	Effect	*SE*/Boot *SE*	*t*	*p*	LLCI	ULCI
Total effect	0.1770	0.0629	2.8156	0.0054	0.0530	0.3011
Direct effect	0.1587	0.0631	2.5152	0.0127	0.0342	0.2831
Indirect effect	0.0183	0.0130			0.0013	0.0551

*Lower and higher conditions are 1 standard deviation below and 1 standard deviation above the mean, respectively. Bootstrapped 95% confidence intervals are derived from 20,000 replications. SE, standard error; CI, confidence interval.*

**FIGURE 2:**
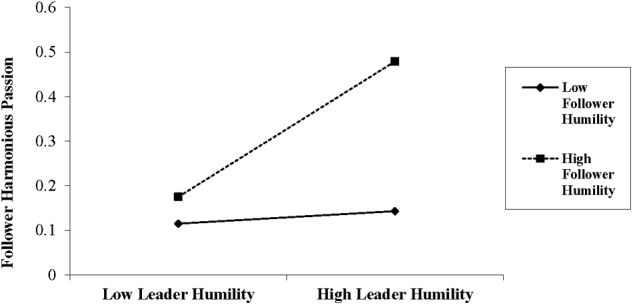
Follower humility as a moderator of leader humility and follower harmonious passion.

The moderating effect of follower humility on the relationship between leader humility and follower harmonious passion is proposed by Hypothesis 2. The Results of model 7 in Table 3 demonstrate the hierarchical regression analysis results of the moderating effect. As shown by the results, the interaction of leader humility (T1) and follower humility (T1) predicts follower harmonious passion (T2) (*β* = 0.07, *p* < 0.10). We then used bootstrapping analysis to further test the moderating effect. As displayed by the results in Table 5, the moderating effect is significant when follower humility is high (1 SD above the mean; effect 0.1497, 95% confidence interval [CI] = 0.0263, 0.2731 [not containing 0]), but not significant when follower humility is low (1 SD below the mean; effect 0.0167, 95% confidence interval [CI] = -0.1006, 0.1341 [containing 0]). By Aiken et al. (1991) method, we drew Figure 2, which visualizes that the positive relationship between leader humility and follower harmonious passion is stronger when follower humility is high (1 SD above the mean) versus low (1 SD below the mean). Therefore, Hypothesis 2 is supported.

**Table 5:** Interactive effect of leader humility and follower humility on follower harmonious passion.

	Effect	*SE*	*t*	*p*	LLCI	ULCI
Low follower humility	0.0167	0.0595	0.2811	0.7789	–0.1006	0.1341
Mean follower humility	0.0832	0.0464	1.7954	0.0742	–0.0082	0.1747
High follower humility	0.1497	0.0626	2.3937	0.0177	0.0263	0.2731

*Lower and higher conditions are 1 standard deviation below and 1 standard deviation above the mean, respectively. Bootstrapped 95% confidence intervals are derived from 20,000 replications. SE, standard error; CI, confidence interval.*

Hypothesis 3 proposes a moderated mediation suggesting that follower humility moderates the indirect positive relation between leader humility and follower performance via follower harmonious passion. The bootstrapping analysis results reported in Table 6 indicate that the moderated mediating effect is significant when follower humility is high (1 SD above the mean; effect 0.303, 95% confidence interval [CI] = 0.0048, 0.0802 [not containing 0]), but not significant when follower humility is low (1 SD below the mean; effect 0.0034, 95% confidence interval [CI] = -0.0200, 0.0364 [containing 0]). Therefore, Hypothesis 3 is supported.

**Table 6:** Interactive effect of leader humility and follower humility on follower performance via harmonious passion.

	Effect	*SE*	LLCI	ULCI
Low follower humility	0.0034	0.0137	–0.0200	0.0364
Mean follower humility	0.0169	0.0128	0.0002	0.0524
High follower humility	0.0303	0.0180	0.0048	0.0802

*Lower and higher conditions are 1 standard deviation below and 1 standard deviation above the mean, respectively. Bootstrapped 95% confidence intervals are derived from 20,000 replications. SE, standard error; CI, confidence interval.*

## Discussion

This paper examines how leader humility affects follower job performance. The analysis results, supporting our proposed model, generated upon self-determination theory, indicate that both a direct positive relationship between leader humility and follower performance and an indirect positive relationship between them, via follower harmonious passion, are found. They also reveal a moderating role of follower humility on the indirect relationship: follower humility strengthens the positive relationship between leader humility and follower harmonious passion and strengthens the indirect relationship between leader humility and follower performance via follower harmonious passion. The findings of this study generate several interesting theoretical and practical implications.

### Theoretical and Practical Implications

Our findings add knowledge to leader humility, passion and self-determination literature in four ways. First, we enrich the understanding of the routes from leader humility to follower performance. Many studies are interested in leader humility’s influence on team outcomes and follower outcomes (e.g., Nielsen et al., 2010; Rego et al., 2017; Sousa and Dierendonck, 2017), but only a few of them give clear evidences of how leader humility works on follower performance. Among those few researches related to the link between leader humility and follower performance, Owens and Hekman’s (2016) explains the effects of leader humility on team performance with a team level mechanism, and Ou et al. (2014) show the influence of a CEOs’ humility on middle managers’ performance with a set of company level procedures, and a cross-level procedure. They somehow leave a gap as to how individual followers process the leader humility to their individual performance. As a contribution to fill that space, our study offers an individual-level mechanism, suggesting that, perceiving leader humility, followers have more harmonious passion at work and hence raise their performance, to further enrich the leader humility effect chain.

Second, we extend leader humility research by considering leadership effectiveness with follower humility, a follower characteristic. The effectiveness of leader humility is influenced by environmental factors, such as top management team faultlines (Ou et al., 2017), leaders’ traits and behaviors other than humility, such as leader narcissism (Owens et al., 2015), and follower factors, such as follower attributions of leader humility (Nielsen et al., 2010). Our study suggests that follower humility is among factors influencing the effectiveness of leader humility, and that a high level of follower humility makes the positive relationship between leader humility and follower harmonious passion at work stronger than a low level of follower humility.

Third, we link leader humility theories and self-determination theory with followers’ harmonious passion, while previous leader humility research focuses more on role modeling effects. Being role models for followers, leaders model ways of pursuing goals (Yaffe and Kark, 2011) and their humble attitudes and behaviors are likely to be emulated by followers (Nielsen et al., 2010; Oc et al., 2015; Mayo, 2017), contributing to follower positive outcomes. We offer an alternative theory, self-determination theory, to explain how leader humility works on follower performance improvement. Humble leaders are not only good models, but also autonomy supporters, which stimulate followers’ harmonious passion.

Fourth, consistent with Liu et al. (2011) research, we find that although harmonious passion is a self-defining characteristic (Vallerand et al., 2003; Forest et al., 2012), the shown level of it can be changed by external autonomy support. This external autonomy support can be not only team-level and higher organizational level autonomy support, found by Liu et al. (2011), but also leader humility, investigated in our study. We suggest that the shown level of follower harmonious passion changes because a part of harmonious passion undiscovered or hidden is stimulated when the follower perceives his/her leader’s high level humility.

Our study has practical implications as well. By examining the interactive effect of leader humility and follower humility, we suggest that humble leaders need the followers who fit them or recognize their virtues to make their leadership more effective. Although not all persons judge humility as a leader virtue (Exline and Geyer, 2004), followers with high humility have a higher level of harmonious passion when they perceive high level leader humility. Therefore, in practice, before assigning employees into different teams, a humility test for them can be run, and besides their task capabilities, the test results are also useful to pair them with different leaders.

Our study also offers a useful management tool, leader expressed humility, to stimulate follower harmonious passion at work. This study shows that follower harmonious passion at work is not independent of leaders’ behaviors. Express humility trainings can be added to organizations’ leader training programs to facilitate leaders to establish humble behaviors that are likely observed by followers, contributing to the generation of followers’ harmonious passion. Consistent with previous research findings that harmonious passion is positively related to performance (Vallerand et al., 2007; Bonneville-Roussy et al., 2011; Ho et al., 2011; Dubreuil et al., 2014; Astakhova and Porter, 2015), in our study, we find that follower harmonious passion partially mediates the positive relationship between leader humility and follower performance. Therefore, expressed humility trainings for leaders are beneficial for follower performance improvement.

### Limitations and Directions for Future Research

Our study has several limitations. First, although we found and logically reasoned the significant relationship between leader humility and follower harmonious passion, we did not test the possible underlying mechanism of this relationship. The focus of our study is the link between leader humility and follower performance, but we did not get into unlimited details in the effect chain. Future research can test our reasoning with felt autonomy support from leaders in the relationship between leader humility and follower harmonious passion or examine other possible psychological mechanism of this relationship. To make a more comprehensive research model, all three kinds of needs in self-termination theory, competence, autonomy and relatedness (Ryan and Deci, 2000), can be involved. Besides, scholars propose that harmonious passion as a motivation is of better quality than intrinsic motivation (Liu et al., 2011), which related to all the three needs (Gagné and Deci, 2005). Future research may control intrinsic motivation to distinguish the effects of harmonious passion.

Second, bootstrapping analyses (using 20,000 bootstrap samples) results support our proposed mediating effect and moderating effect, while hierarchical regression analyses results indicate that both the mediating effect of follower harmonious passion on the relationship between leader humility and follower performance, and the moderating effect of follower humility on the relationship between leader humility and follower harmonious passion are marginal significant. Therefore, we suspect the marginal significance may be related with our sample size (*N* = 200), which is not a big one. Future research may test our model with more participants.

Third, we use self-determination theory to test the mediating role of follower harmonious passion, but there is another kind of work passion, obsession passion, being proved to be related with performance (Bélanger et al., 2013; Omorede et al., 2013; Astakhova and Porter, 2015). Scholars state that in some conditions, such as being under pressure, harmonious passion has a stronger effect on performance (Vallerand et al., 2003), while in some other conditions, such as being exposed to failure information, obsession passion is more effective than harmonious passion (Bélanger et al., 2013). Although harmonious passion and obsession passion work on performance in different ways, there may be a link between leader humility and follower performance via follower obsession passion. Some interesting findings may be found by future researches testing both the harmonious passion path and the obsession passion path.

Fourth, expressed humility has three dimensions: willingness to view oneself accurately, appreciating others’ strengths and contributions, and being open to feedbacks, advices and new ideas (Owens et al., 2013). However, we investigated it as a whole without examining whether leaders’ three kinds of humble behaviors affect follower harmonious passion in different ways or through different mechanisms. For example, a leader with humility can express his/her autonomy support to followers not only by offering support as a significant person in workplace, but also by building a support environment in the team as a team leader who has influences on the majority of team members. To test whether the three dimensions of leader expressed humility function differently in these two ways, a multi-level study can be conducted in the future.

Fifth, this study tries to discover followers’ psychological mechanism, which leads us to focus on followers’ perception of leader humility and their own judgments of their own humility. We therefore measured both leader humility and follower humility with follower reports. As proposed by several scholars, though, one’s self-rated humility may be different with an other-rated one due to self-serving biases and the nature of humility (Davis et al., 2010; Rego et al., 2017). Future studies, when focusing on self-reported or other-reported humility, may control the other one. Besides, future studies bringing both self-reported and other-reported humility into research models may collect some interesting findings. If researchers are worried that most followers behave humbly in front of their leaders, followers’ humility can be rated by their coworkers.

## Conclusion

The purpose of our study is to examine the effect of leader humility on follower performance. This study finds that leader humility contributes to follower performance improvement, and follower harmonious passion partially mediates this positive relationship. The influencing process is moderated by follower humility: when followers are of high level of humility leader humility has a stronger influence on follower harmonious passion, and then on follower performance, than when followers are of low level of humility. As an exploring study to investigate the association between leader humility and follower performance with a self-determination mechanism, this study highlights the link between leaders’ humble behaviors and followers’ work passion.

## Ethics Statement

This research was carried out in accordance with the ethical guidelines of the American Psychological Association in protecting participants’ wellbeing and confidentiality. The research was approved by the National Natural Science Foundation of China. By the cover pages of questionnaires and the introduction talk of the research, all subjects were informed of the research purposes, their freedom of participating and quitting the research, and the assurance of the confidentiality.

## Author Contributions

LS proposed the research. HD drafted the manuscript. HD and YW carried out the data analysis. All authors contributed to the research model improvement, participated in the data collection and manuscript revision, read and approved the submitted version.

## Conflict of Interest Statement

The authors declare that the research was conducted in the absence of any commercial or financial relationships that could be construed as a potential conflict of interest.
